# Clinical Characteristics of Presenile Cataract: Change over 10 Years in Southern Taiwan

**DOI:** 10.1155/2021/9385293

**Published:** 2021-03-20

**Authors:** Hun-Ju Yu, Ming-Tse Kuo, Pei-Chang Wu

**Affiliations:** ^1^Department of Ophthalmology, Kaohsiung Chang Gung Memorial Hospital and Chang Gung University College of Medicine, Kaohsiung, Taiwan; ^2^Graduate Institute of Clinical Medicine, College of Medicine, Kaohsiung Medical University, Kaohsiung, Taiwan

## Abstract

**Purpose:**

The purpose of this study is to investigate the clinical characteristics of presenile cataract and compare that to ten years ago in southern Taiwan.

**Methods:**

The subjects who received cataract surgeries aged 30 to 54 years were recruited in Kaohsiung Chang Gung Memorial Hospital during September 2015 and August 2016. Patients with uveitis or those who received combined cataract surgeries were excluded. Retrospective chart review was performed in this study.

**Results:**

A total number of 2439 cataract surgeries were performed, and 302 (12.38%) eyes were having presenile cataract. Mean age was 47.55 ± 5.64 years old, and mean axial length was 26.00 ± 2.89 mm. Among 302 presenile cataract eyes, the leading cause was high myopia (defined as mean axial length ≥ 26 mm, 47.02%), followed by diabetes mellitus (26.82%). In types of lens opacity analysis, 67.55% of the patients were nuclear sclerosis dominant. Compared to the previous study 10 years ago, the leading cause of presenile cataract changed from idiopathy to high myopia, whereas the lens opacity types changed from posterior subcapsular opacity dominant to nuclear sclerosis dominant.

**Conclusions:**

High myopia has become the most important clinical characteristic associated with presenile cataract in a myopia epidemic area, and the most common type of lens opacity was nuclear sclerosis. With the increasing prevalence of high myopia, we should pay more attention to the management of presenile cataracts in high myopes to avoid complications.

## 1. Introduction

Worldwide, cataract remains the leading cause of blindness and the second leading cause of moderate and severe vision impairment [1]. Nearly half of visual impairment is attributed to cataract in Taiwan [2], which is much higher than the rest of the world. The risk factors of cataract formation may be multifactorial, including age, genetic factors, cigarette smoking, sunlight exposure, diabetes mellitus (DM), hypertension, steroid use, and systemic medications [3, 4]. Among them, age is still the single most important risk factor for cataract. It appears that cataract is being found in younger patients in Taiwan [5]. Few studies have investigated the risk factors for presenile cataracts [6–9]. Praveen et al. showed that the risk factors associated with cataract in young individuals were atopy, idiopathy, high myopia, steroid usage, sunlight exposure, and DM in India [6]. Atiya Rahman et al. found that idiopathy, DM, high myopia, and smoking contributed to presenile cataract in Pakistan [7]. Park et al. pointed out that DM and hypertension were associated with young age cataract in Korea [8]. Our previous study reported that the main etiologies of presenile cataract were idiopathy, DM, and high myopia [9]. We believe that it is worth investigating presenile cataract in myopia epidemic countries as younger age of cataract surgery was performed.

In Taiwan, patients less than 55 years old who need cataract surgery receive medical claims reviewed by National Health Insurance. In order to understand the clinical features of presenile cataract, we reviewed the medical records and compared them to our previous study ten years ago. Identifying the frequency and changes of these risk factors will allow for better management of presenile cataract in the future and could help avoid younger onset age of cataract.

## 2. Patients and Methods

### 2.1. Study Populations

This retrospective chart review comprised of consecutive patients aged less than 55 years old who received cataract surgery after medical claims reviewed by National Health Insurance during September 2015 and August 2016 at a single tertiary medical center in Kaohsiung Chang Gung Memorial Hospital in Taiwan. An institutional review board (IRB)/Ethics Committee approval was obtained from the Committee of Medical Ethics and Human Experiments of Chang Gung Memorial Hospital (CGMH, Taiwan, IRB No. 201601279B0), and the tenets of the Declaration of Helsinki were followed. The IRB waived the requirement for informed consent.

In our previous study, the medical records of all patients aged 54 years or younger (163 patients) who had undergone cataract surgery at the Chang Gung Memorial Hospital, Kaohsiung, were retrospectively reviewed [9]. Patients who had undergone previous vitreoretinal procedures or who had suffered from congenital cataracts were excluded from study participation. All cases suspected of being associated with steroid-induced cataracts were also excluded from this study. Patient age, gender, eye axial length (AXL) measurement, ocular and systemic comorbidities, and the type of cataract were recorded. Each group was divided into idiopathic, diabetic, and highly myopic subgroups based on the etiology of each specific cataract.

### 2.2. Grading of Lens Opacity and AXL Measurement

Those who had congenital cataracts, severe trauma (eyeball rupture), refractory uveitis, and combined vitreoretinal surgeries were excluded. After pupil dilatation, the eyes were graded at the slit lamp using the Lens Opacities Classification System III by experienced ophthalmologists for nuclear sclerosis (NS), cortical opacity (CO), posterior subcapsular opacity (PSCO), and mature cataracts. Standard lens photographs were taken using a Haag-Streit BX 900 Photo Slit Lamp. The AXL was measured in each eye using partial coherence interferometry (IOLMaster, Carl Zeiss Meditec AG). The criterion for the selection of high myopic eyes was AXL 26 mm or more to avoid myopic shift. Patient age, gender, lens opacity type, AXL, myopia diopter, ocular surgery history, steroid usage, trauma, and systemic disease including DM and hypertension were recorded.

### 2.3. Statistical Analysis

Statistical significance was defined as *P* < 0.05. Student's *t*-test and analysis of variance were used in the analysis. All data analyses were performed using JMP software (version 9.0.0; SAS Institute, Cary, NC).

## 3. Results

**Figure 1:**
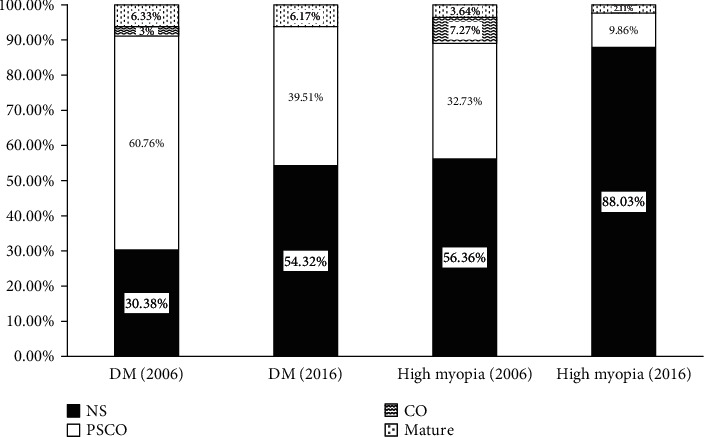
Changes of incidence in lens types for presenile cataract in 10 years.

**Figure 2:**
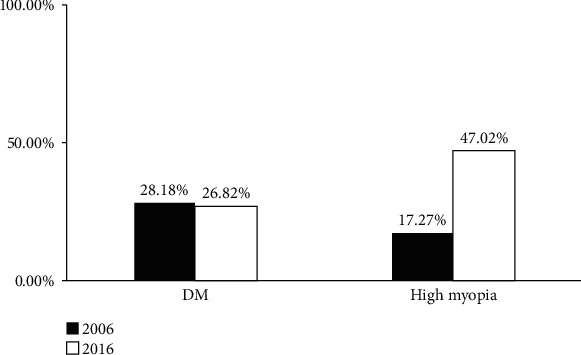
Changes of incidence in DM and high myopia for presenile cataract in 10 years.

During September 2015 and August 2016, a total number of 2439 cataract surgeries were performed and 302 (12.38%) eyes were having presenile cataract which were recruited into our study. 51.33% were male and 48.67% were female (Table 1). The mean age was 47.6 ± 5.6 years old, and the mean AXL was 26.00 ± 2.89 mm. Among 302 presenile cataract eyes, the leading cause was high myopia (defined as AXL ≥ 26 mm, 47.02%), followed by DM (26.82%), ocular surgery history (26.16%), and hypertension (21.52%). In types of lens opacity analysis, 67.55% of patients were NS dominant and 25.17% of patients were PSCO dominant. Among the patients with high myopia, 88.03% were NS dominant. Of the patients with DM, 54.32% were NS dominant (Figure 1). Compared to the previous study 10 years ago, the leading risk factor of presenile cataract changed from DM to high myopia (Figure 2), whereas the lens opacity types changed from PSCO dominant to NS dominant (Figure 1).

The age of performing cataract surgery among those patients with DM without panretina photocoagulation (PRP), those patients with DM post-PRP, or those patients with DM post-PRP and vitrectomy (trans-pars plana vitrectomy, TPPV) was not significantly different (*P* = 0.557). The severity of DM did not get younger in age for receiving cataract surgery.

There were 14 patients that received laser-assisted *in situ* keratomileusis (LASIK) before cataract surgery. 12 of them were having high myopia. There was no significant difference in age and AXL between patients with and without LASIK before cataract surgery in myopia patients (Table 2). In high myopia patients, there was also no significant difference in age and AXL between patients with and without LASIK before cataract surgery (Table 3).

## 4. Discussion

The prevalence of myopia is increasing and has become an important issue in public health [10, 11]. In Taiwan, the prevalence of myopia is 84% in high school students and approximately 24% of the myopic population become high myopes as adults [12]. One of the complications of myopia is presenile cataract. Even though presenile cataract in high myopes can be treated through cataract surgery, high myopia is a significant risk factor for intraoperative complications such as posterior capsule rupture and development of retinal detachment or after-cataract after cataract surgery [13–22]. The increasing prevalence of high myopia explained why high myopia became the leading cause of presenile cataract in this study, whereas it was the third cause 10 years ago. Thus, myopia control for high myopia prevention will become more important in the future.

The reason why myopia leads to early-onset cataract is still not well-understood. We hypothesize that it may be caused by vitreous microtrauma to the lens, whereas high myopia leads to vitreous liquefaction and zonula degeneration that induces more microtrauma to the lens and earlier cataract formation.

In types of lens opacity analysis, nuclear sclerosis is predominant in the high myopia group, which was the same as compared to ten years ago. In DM group, the main lens opacity type changed from PSCO to NS when compared to the previous study. The increasing prevalence of high myopia might explain the result and indicated that more DM subjects also had high myopia leading to more NS-type cataracts.

The number of people with DM is increasing, and the global prevalence was estimated to be 2.8% in 2000 and is expected to reach 4.4% by 2030 [23, 24]. Cataract is considered a major cause of visual impairment in diabetic patients as the incidence and progression of cataract are elevated in patients with DM. Also, clinical studies have shown that cataract development occurs at an earlier age in diabetic patients compared to nondiabetic patients [25]. The pathogenesis of diabetic cataract development is still not fully understood. Overall, up to 20% of all cataract procedures are estimated to be performed for diabetic patients. In our study, 26.82% patients had DM while receiving cataract surgery in young age before the age of 55, which is similar to that in general cataract surgery population.

Regarding the risk factors for cataracts in diabetic patients, impaired fasting glucose is considered for the development of cortical cataract in some studies [23]. There is no previous study that showed the relationship between cataract and diabetic retinopathy severity. We subgrouped the diabetic retinopathy severity in three groups: no diabetic retinopathy, proliferative diabetic retinopathy postphotocoagulation only, and postvitrectomy. The age of cataract surgery had no statistical difference among these three groups. The result implicated that getting diabetes or not is the most important issue of diabetic cataract, but not the following blood sugar control or diabetic retinopathy severity. It cannot be overstated that diabetes prevention is better than a cure.

LASIK has gained popularity as an effective means of correcting refractive errors. Because patients who had LASIK in the early 1990s have been aging, the number of patients requiring cataract surgery after LASIK has been increasing. Regarding patients having cataract surgery after LASIK, Iijima et al. showed that the patient age at the time of cataract surgery was significantly younger (10 years old) in patients post-LASIK than AXL-matched eyes without LASIK [26]. Our study showed that there was no significant difference in age and AXL between patients with and without LASIK before cataract surgery, both in myopia patients and high myopia patients. The difference between our study and that of Iijima et al. is that our study group is younger than 55 years old and had less study cases. Further studies are needed to investigate whether or not cataract development will occur at an earlier age in a post-LASIK population.

## 5. Conclusion

In conclusion, high myopia has become the most important clinical characteristic associated with presenile cataract, and the most common type of lens opacity was nuclear sclerosis in our study. With the increasing prevalence of high myopia, we should pay more attention in managing presenile cataract in high myopes to avoid further complications.

## Figures and Tables

**Table 1:** Patient demographics.

Factor	*N*	%
Male	155	51.3%
Female	147	48.7%
Axial length (AXL)		
<26 mm	160	53.0%
≥26 mm (high myopia)	142	47.0%
Diabetes mellitus (DM)	81	26.8%
Without PRP	27	33.3%
Post-PRP only	28	34.6%
Post-PRP and TPPV	26	32.1%
Hypertension	65	21.5%
Other systemic disease	56	18.5%
Ocular surgery	79	26.2%
Post-TPPV	60	76.0%
Post-LASIK	14	17.7%
Post-SE	3	3.8%
Others	2	2.5%
Ocular trauma history	18	6.0%
Steroid usage	5	1.7%
Lens type		
NS	204	67.6%
PSCO	76	25.2%
CO	8	2.7%
Mature	14	4.6%

^∗∗^PRP: panretinal photocoagulation; TPPV: trans-pars plana vitrectomy; LASIK: laser-assisted *in situ* keratomileusis; SE: scleral buckle; NS: nuclear sclerosis; PSCO: posterior subcapsular opacity; CO: cortical opacity.

**Table 2:** Age and axial length (AXL) difference between eyes with/without LASIK in myopia patients.

	Myopia patients post LASIK (*N* = 14)	Myopia patients without LASIK (*N* = 177)	*P* value
Age	47.5 ± 1.5	47.1 ± 0.4	0.786
AXL	28.57 ± 0.67	27.43 ± 0.19	0.104

^∗^LASIK: laser-assisted *in situ* keratomileusis.

**Table 3:** Age and axial length (AXL) difference between eyes with/without LASIK in high myopia patients.

	High myopia patients post-LASIK (*N* = 12)	High myopia patients without LASIK (*N* = 130)	*P* value
Age	47.8 ± 3.2	46.7 ± 5.9	0.530
AXL	29.15 ± 1.94	28.45 ± 2.10	0.267

^∗^LASIK: laser-assisted *in situ* keratomileusis.

## Data Availability

The data used to support the findings of this study are included within the article.
